# Narratives of preterm and full‐term preschool‐aged children: Analyses of different narrative dimensions

**DOI:** 10.1111/bjdp.12555

**Published:** 2025-03-16

**Authors:** İbrahim Akkan, Şeref Can Esmer, Işıl Doğan, Aslı Aktan‐Erciyes, Ö. Ece Demir‐Lira, Tilbe Göksun

**Affiliations:** ^1^ Koç University Istanbul Turkey; ^2^ Yale University New Haven Connecticut USA; ^3^ University of California, Davis Davis California USA; ^4^ Kadir Has University Istanbul Turkey; ^5^ University of Iowa Iowa City Iowa USA

**Keywords:** narrative generation, neonatal status, preterm, storytelling

## Abstract

Preterm birth increases the likelihood of early language and cognitive delays, but less is known about later aspects of language development, such as narrative generation. Narrative skills involve dimensions, such as linguistic and narrative complexity, and preterm (PT) and full‐term (FT) children's narrative performances may vary across these dimensions. We investigated the role of neonatal status on the total number of words produced, linguistic complexity, and narrative complexity across two presentation modes: narrative generation while seeing pictures and narrative generation after watching an animated video. Seventy‐one Turkish‐reared preschool‐aged children (31 PT [*M*_age_ = 48.70, SD = 1.53] and 40 FT [*M*_age_ = 48.83, SD = 1.63]) participated in the study. Despite having lower expressive vocabulary skills (assessed by a standardized task) than full‐term children, preterm children performed comparably in both picture and animated video‐stories, except PT children tended to produce longer narratives in the picture story, possibly due to the different demand characteristics of the tasks. Overall, our findings support the possibility of interacting factors that may help PT children overcome challenges in narrative development.

**Key points**

**What is already known on this subject?**

- Preterm birth (i.e., gestational age <37 weeks) is associated with an increased likelihood of developmental delays in language and cognitive skills.
- A focus on narratives is vital in that narrative generation is related to subsequent gains in academic achievement.
- Previous studies show that dimensions of narratives such as linguistic elements or story elements might be affected children's neonatal status.
- Narrative generation might also vary as a function of the presentation mode of the narrative task.

**What does the present study add?**

- This study examines whether preterm (PT) and full‐term (FT) children exhibit different performance trends in using linguistic elements (e.g., total number of words, linguistic complexity) or integrating linguistic and story elements (e.g., narrative complexity).
- Additionally, we investigate whether narrative presentation modes (picture‐story, animated video‐story) elicit performance differences due to varying task requirements.
- The present study differs from the earlier work on narrative skills for its focus on narrative generation, as opposed to narrative retelling.

Preterm birth (i.e., gestational age < 37 weeks) is associated with an increased likelihood of developmental delays in language and cognitive skills (Foster‐Cohen et al., 2007; Goldenberg et al., 2008; Sansavini et al., 2014). Due to its complex nature and requirement for efficiency in both linguistic and cognitive skills (Berman, 1997), children's narrative performance may also be linked to their neonatal status. A focus on narratives is vital in that narrative generation is related to subsequent gains such as reading comprehension (Griffin et al., 2004), academic achievement (Fazio et al., 1996), performance on norm‐referenced language assessments (Ebert & Scott, 2014), and social competence (von Klitzing et al., 2007). Variability in narrative generation might be differentially linked to the dimensions of narrative skills (e.g., using linguistic elements or integrating linguistic and story elements, i.e., narrative complexity). Narrative generation might also vary as a function of the presentation mode of the narrative task (e.g., a picture‐story vs. an animated video‐story). This study examines whether preterm (PT) and full‐term (FT) children exhibit different performance trends in using linguistic elements (e.g., total number of words, linguistic complexity) or integrating linguistic and story elements (e.g., narrative complexity). Additionally, we investigate whether narrative presentation modes (picture‐story, animated video‐story) elicit performance differences due to varying task requirements.

### Preterm birth

In terms of their gestational age, preterm births are classified as extremely PT (gestational age < 28 weeks), very PT (28 weeks < gestational age < 32 weeks), and moderate or late PT (32 weeks < gestational age < 37 weeks) (WHO, 1977). The risks of mortality and morbidity are higher in early gestation (<34 weeks) and late preterm birth (34–37 weeks) is encountered more frequently (Saigal & Doyle, 2008). Although the survival rates have increased because of technological advances, lower birth weight and lower gestational week are linked to the increased likelihood of neuropsychological delays in PT children (Allotey et al., 2018; Brydges et al., 2018; Cheong et al., 2017; Kobaş et al., 2023; Snijders et al., 2020). For example, Cheong et al. (2017) reported general cognitive and language delays on a norm‐based battery in 2‐year‐old PT children, most marked in the language domain. While most studies report preterm birth‐related delays and an increase in the gap between PT and FT children as they age (Sansavini et al., 2011), other moderating factors might partially influence the gap between children or lead PT children to catch up with their FT peers in language and cognitive domains, especially in later years where complex language skills emerge (DeMaster et al., 2019).

Development is cumulative, and gains build over time (Iverson, 2021). Potential delays in early motor skills among PT infants can lead to relatively lower language scores due to cascading effects on object exploration and limited language learning opportunities compared to FT infants (Kobaş et al., 2023), but development remains continuous. As parents actively engage with their infants and create enhanced learning opportunities in the environment, it generates cascading effects that assist PT infants in catching up with their FT peers. The developmental cascades approach (Oakes, 2023; Oakes & Rakison, 2019) offers an avenue for explaining how PT children may either catch up with their FT peers or fall behind in language skills. According to this approach, multiple pathways (i.e., initial skills interacting with other factors) can lead to the same outcome through adaptive features of development. Similar to sociocultural factors, neonatal status might be associated with diversity in developmental pathways. Furthermore, higher brain plasticity in PT infants, resulting from interrupted pregnancies during the critical period where rapid changes occur in brain development, may allow PT children to benefit more from various environmental factors, such as parental language input (I. Doğan, M. Kobaş, E. Kızıldere, A. Aktan‐Erciyes, E. Demir‐Lira, I. Akman, & T. Göksun, unpublished data; Esmer et al., 2024), and higher parental education (Loeb et al., 2020) enabling them to catch up with their FT peers in vocabulary development (Demir‐Lira & Göksun, 2024). However, prior research on cascading effects of PT birth primarily focused on vocabulary development at earlier ages (I. Doğan, M. Kobaş, E. Kızıldere, A. Aktan‐Erciyes, E. Demir‐Lira, I. Akman, & T. Göksun, unpublished data; Esmer et al., 2024). This study asks how PT birth would interact with narrative development, a later developing and integrative language skill?

### Narrative development

In a structured narrative, events are retold and evaluated from the speakers' perspective, structured into chronological and causal sequences of sub‐events (Bruner, 1990; Crawshaw et al., 2020; Labov & Waletzky, 1967). These structures are organized according to narrative schemas, which allow speakers to mentally arrange and connect these events (Merritt & Liles, 1989; Pratt et al., 1989; Stein & Glenn, 1979). Moreover, narratives are structured at microstructure and macrostructure dimensions (Ninio & Snow, 1996). The microstructure dimension comprises linguistic analyses such as vocabulary and grammar proficiency, total number of words, type‐token ratio, and linguistic complexity. The macrostructure dimension, on the other hand, focuses on the pragmatic analyses of story elements, such as setting, characters, initiating an event, action, consequence, and ending. In a coherent narrative, microstructure elements such as lexical diversity and linguistic complexity are intertwined with the story elements. Narrative complexity (Gillam & Pearson, 2017) assesses how children integrate microstructure and macrostructure dimensions to produce complete narratives with time‐ordered causal events using necessary linguistic elements. However, it fails to capture the microstructure dimension, such as the narrative length or the sentences' complexity, especially in Turkish. While causality is a macrostructural component, in Turkish, it is often expressed through microstructural linguistic devices such as morphological suffixes (e.g., *gül‐DÜr*, the verb *gül* ‘laugh’ is causativized with the causative marker ‐*DÜr* to produce *gül‐DÜr* ‘make laugh’). Thus, it is essential to consider the structure of the language when investigating narrative development.

The period from age 3 to 6 is a relevant age range to observe variations in narrative abilities (Stites & Özçalışkan, 2017). At age 5, children's narratives become more structurally complex but still show individual variability (Hudson & Shapiro, 1991). They begin to organize their narratives around goals and attempts and mostly refer to the main components, such as initial orientation, complication, and resolution (Berman & Slobin, 1994; Reilly, 1992). Yet, the narratives may lack some components, such as failing to include an ending (see Peterson & McCabe, 1983; Reese et al., 2011). Therefore, preschool‐aged children present an ideal group to investigate the role of different factors on narrative development.

### Narrative development in PT children

In complex tasks such as generating well‐structured narratives, PT children might not catch up with their peers' narrative performance. The benefit of higher brain plasticity due to PT birth may not extend to more complex language tasks (i.e., narrative generation), as argued in another case of high plasticity, prenatally brain‐injured children (Demir et al., 2010, 2014; Demir‐Lira & Göksun, 2024). Supporting this argument, earlier studies with older age groups showed differences in PT and FT children's narratives (Crosbie et al., 2010; Stipdonk et al., 2020), but only in the microstructure dimension. For example, Crosbie et al. (2010) found that PT children aged 9–10 had difficulties formulating complex sentences, albeit producing similar narratives regarding the story elements (i.e., initiating an event, action, consequence, and ending). Stipdonk et al. (2020) reported similar results with children aged 10. They found a significant difference in linguistic complexity but not in story elements. Interestingly, only PT children's mean composite *z*‐score of story element scores was higher than those mean *z*‐scores of general language scores (e.g., vocabulary knowledge and sentence formulation). This finding indicates that PT children might have difficulties with vocabulary knowledge or sentence formulation, but they may overcome these difficulties in more real‐life scenarios, such as narrative generation. Together, these findings may attest to the challenging nature of narrative tasks, which engage several aspects of cognitive and linguistic abilities, highlighting the differential performance trends of children on different developmental trajectories, such as PT children. Therefore, it is important to investigate different dimensions of the narratives in PT children.

### Narrative generation in different tasks

Narratives are generally elicited using a generation task, in which children produce narratives without previous exposure to the story (e.g., Ebert & Scott, 2014), or a retelling task in which the experimenter tells a story, and the child retells it (Gillam & Pearson, 2004). Narrative retelling depends on the extent to which children can use the linguistic input they receive, while narrative generation may reflect what children can generate themselves without an example. Narratives can be presented by verbally telling a story (e.g., Merritt & Liles, 1987), a series of pictures (e.g., Bishop & Donlan, 2005), silent videos, or a combination of the two, such as videos with verbal narratives (e.g., Gazella & Stockman, 2003). Several studies have indicated an influential effect of the presentation mode on the quantity (e.g., length) and quality (e.g., linguistic complexity) of the narratives (Diehm et al., 2020; Duinmeijer et al., 2012; Klop & Engelbrecht, 2013; Schneider & Dubé, 2005). For example, in a narrative retelling task, children following typical developmental pathways between the ages of 3–5 produced longer narratives with more diverse vocabulary and an increased proportion of complex sentences in a video story as opposed to a picture format, but no difference was found for story elements (Diehm et al., 2020). Similarly, typically developing children aged 5–7 narrated more story elements when presented with audiovisual as opposed to audio or visual stimuli only (Schneider & Dubé, 2005). On the other hand, Klop and Engelbrecht (2013) found that typically developing children in third grade produced comparable narratives in response to wordless picture books and animated videos. These results may emphasize the advantages of presenting video stories with verbal narratives for the microstructural dimension in narrative retells among 3‐ to 7‐year‐olds, although this effect may diminish as children grow older.

Performance differences observed across presentation modes might be due to varying task demands generated by different narrative presentation modes. In multimedia presentation mode, processing multiple pieces of information may increase cognitive load, resulting in the production of fewer complex sentences that contain more story elements (but see Diehm et al., 2020). Because of the rich information they receive, instead of producing complex sentences, they may focus on conveying that information and tend to produce simpler sentences. Although performance differences in narratives are investigated in retell conditions, no study investigated them in generation tasks and compared atypically and typically developing children. Therefore, it is essential to see how children following possibly different developmental trajectories (i.e., PT children) generate narratives in response to animated video‐story and picture‐story presentation modes.

### The present study

We investigated the role of neonatal status on narrative generation at age 4, considering dimensions of narratives: total number of words, linguistic complexity, and narrative complexity scores. Focusing on these early years is important because children exhibit great variability in their narrative generation during these ages, and there is a lack of literature on earlier stages of narrative development. Children's language skills at the age of 4 are still developing; thus, we argue that documenting skills at these early developmental stages is important for monitoring skill progression and pinpointing the divergence/convergence of skills over the course of development, both within and between PT and FT children. We examined narrative generation across two narrative presentation modes, a picture‐story and an animated video‐story, to see whether PT and FT children would perform differently across the two presentation modes with diverse demands.

We first asked whether PT and FT children differed in their general expressive language scores; we hypothesized that (1) PT children would have lower expressive language scores than their FT peers, as earlier studies reported (e.g., Stipdonk et al., 2020). Second, we asked whether PT children differed from their FT peers regarding their narrative performance across narrative dimensions. Regarding this question, we proposed a non‐directional hypothesis: (2a) One possibility could be lower performance in all scores of the narrative performance. Demir et al. (2010) argue that the benefits of high plasticity in brain‐injured children may not extend to complex language skills, such as narrative performance. We contend that this may also apply to preterm (PT) children, who exhibit high plasticity due to early birth (Demir‐Lira & Göksun, 2024). Alternatively, (2b) PT children would perform worse in the total number of words and linguistic complexity but similarly in narrative complexity compared to their FT peers, as Stipdonk et al. (2020) showed that PT children aged 10 performed worse in linguistic (i.e., linguistic complexity) elements but not in story elements. Third, we asked whether the difference, if any, between PT and FT children in their narrative performance was the same across picture‐story vs. animated video‐story presentation modes, which may have different demand characteristics. We cannot form an a priori hypothesis regarding presentation mode due to contradictory results from earlier studies in narrative‐retell tasks (e.g., Diehm et al., 2020; Klop & Engelbrecht, 2013) and no study investigating them within narrative generation tasks. However, we hypothesized that there would be a difference in the linguistic complexity of the narratives between PT and FT children for the animated video‐story mode but not for the picture‐story mode, as the animated video‐story may be richer in information and may affect PT's sentence formulation more than their FT peers.

## METHOD

### Participants

The present study was part of a longitudinal study investigating PT and FT children's cognitive and language development between 12 and 48 months at four time points. Participants were recruited through a local hospital's paediatric services database, a rehabilitation center, and social media ads. This study focused on the data from the last time point when children were 4 years old. The demographic information for each group is presented in Table 1. The final sample consisted of 38 boys and 33 girls, in total 71 Turkish‐reared preschool‐aged monolingual children (*M*_age_ = 48.76 months, SD = 1.63, range: 46.4–53.4 months), 31 PT (12 girls; *M*_age_ = 48.70, SD = 1.53) and 40 FT (21 girls; *M*_age_ = 48.83, SD = 1.63). However, not all children completed all tasks due to boredom and fatigue. In total, there were 67 children (29 PT, 28 FT) for the expressive language measure, 64 children (29 PT, 35 FT) for the picture‐based task, and 47 children for the cartoon‐based task (20 PT, 27 FT). The study was approved by Koç University's Institutional Review Board (Project name: A longitudinal study examining preterm and full‐term infants' early language and cognitive development. Protocol no: 2018.118.IRB3.083).

**TABLE 1:** Descriptive statistics of the variables.

	Preterm			Full‐term			*t*	*p*
	*N*	*M*	SD	*N*	*M*	SD		
Age	31	48.70	1.53	40	48.83	1.63	0.34	.737
Birth weight	31	1475.81	561.48	40	3378.75	405.23	15.93	<.001
Gestation week	31	30.13	3.16	40	39.28	1.40	15.02	<.001
Maternal education	30	15.63	2.74	40	16.63	2.24	1.67	.100
Expressive vocabulary	29	50.14	12.44	38	56.18	9.89		
Picture‐story total number of words	29	45.90	19.23	35	36.43	15.00		
Picture‐story linguistic complexity	29	0.24	0.21	35	0.28	0.19		
Picture story narrative complexity	29	4.38	1.64	35	3.71	1.22		
Animated video‐story total number of words	20	55.15	30.25	27	44.48	18.28		
Animated video‐story linguistic complexity	20	0.22	0.13	27	0.34	0.25		
Animated video‐story narrative complexity	20	3.80	1.54	27	3.52	2.06		

*Note*: Narrative complexity scores have a minimum of 0 and a maximum of 12.

Abbreviations: *M*, mean; *N*, sample size; SD, standard deviation.

### Measures

#### Turkish expressive and receptive language test—expressive subtest (TIFALDI; Berument & Güven, 2013)

TIFALDI expressive subtest (picture‐vocabulary task) was used to assess children's expressive vocabulary knowledge. This norm‐based test is used for children between the ages of 2 and 12. It includes 80 items. The items are presented in blocks of 8, including items likely to be known by an age group. The tester starts with the block corresponding to the child's age and continues with the subsequent block. Within the test, children are presented with pictures belonging to semantic categories such as objects, animals, foods, and clothes, and they are asked to report the names of the items. The block where the child correctly answers the previous eight items is counted as a base score. The test ends at the block where the child fails six items within a set of eight questions. We calculated raw scores by summing the base score and the number of correct answers before the ending point.

#### Picture‐story (test of narrative language—second edition [TNL – 2], Gillam & Pearson, 2017)

The Late for School and the shipwreck stories were used to assess children's narrative performance. The first story was about a child being late for school and missing the bus; the second story was about a girl constructing a ship for the school assignment and dropping it before going to school. Each story included five pictures.

#### Animated video‐story—Oktapodi (Bocabeille et al., 2007)

A cartoon unfamiliar to Turkish‐speaking children was used to assess children's narrative performance with a dynamic stimulus. Children watched a series of humorous events about two octopi that escaped from a stubborn restaurant cook in the streets of a small Greek village. The video was 1.5 min long.

### Procedure

After obtaining informed consents and collecting demographic information from parents, parent–child dyads participated in online Zoom sessions with experimenters sharing their screens with presentations. Demographic information^1^ included maternal education, children's gestation week and birth weight, and we asked parents to fill out this information. The experiment was conducted in two sessions; the second session took place on average 10 days after the first. Within the first session, children completed the narrative generation tasks, which took 10–15 min. They completed the expressive vocabulary test in the second session, which took approximately 10 min. They had to produce two different stories for the picture‐story generation. They were shown a five‐picture frame for each story on a computer screen without text or sound and asked to generate a story that the pictures depicted. The administration differed from the standardized methods to convert the data collection to an online format. Contrary to standardized administration, the experimenter did not provide an example of story generation. Instead, we asked children to look at the pictures and generate the story that the pictures described after presenting each story's pictures. Once the children had looked at all the pictures, the experimenter asked them to generate a story while the pictures were present in their view. The exact prompt was, “Now I will show you some pictures. These pictures tell a story. I want you to sift through each picture, one by one, and then tell me what story the pictures tell.” After the picture story task, children watched a wordless cartoon about two octopi for 1.5 min and generated the story after the cartoon ended. Children were told that the experimenter never watched the cartoon before and was curious about what happened in the cartoon. The prompt was, “Now we are moving on to a very nice cartoon game. I will show you a cartoon now, but since only children can watch that cartoon, I will not be able to watch it. I have never watched it before. What I want from you is that since I have not watched this cartoon, you watch it carefully and then tell me and your mother about it. Everything that happened in the cartoon from beginning to end.” Parents were asked to stay quiet and not interrupt children while they generated the stories. For both tasks, if the child had stopped narrating, the experimenter would have said “Is that it?” For each task, the trials ended when children reported that they had nothing else to add. If the children had stated they would not want to complete the task, the task would have been skipped.

### Coding

#### Speech

Children's narratives and parents' and experimenter's prompts were transcribed verbatim from Zoom recordings to code children's narrative scores and to rule out the effect of getting help from the parent or the experimenter's prompts. Sometimes, experimenters used “then what happened?” or “what happened after that” when children stopped talking, or parents tried to help children by providing details. The details elicited after such prompts by the experimenter or parents were removed from the transcriptions and not taken into account for scoring. Children's narratives were transcribed based on Berman and Slobin's criteria (1994). The unit of transcription was the utterance in which any word was preceded and followed by a pause; within an utterance, we included all filled pauses (e.g., uhm). As we were only interested in the part where children narrated stories, other parent–child conversations and parents' interruptions were excluded from the calculations. Another research assistant checked and corrected each transcription. Each video recording was rewatched and corrected; the inaudible or vague parts were not entered.

#### Dimensions of narratives

##### Total number of words

We counted the total number of word tokens and the number of clauses in the story. A word token is any word included in the story (a word is counted five times if it is said five times). Only clauses including a predicate that contributed to the story were included in the analyses (i.e., side comments, e.g., ‘I want to sleep [Uyumak istiyorum],’ were not counted).

##### Linguistic complexity

Linguistic complexity scores were calculated using the criteria in earlier studies (Kızıldere et al., 2020, 2022). First, transcriptions were formed into one verbed clause per line using the definition of a clause, any unit that contains a unified predicate expressing a single activity, event, or state (Berman & Slobin, 1994). Predicatives are the elements of a clause predicate that supplement the subject or object through a verb. Simple sentences were coded as clauses with only one predicate. Complex sentences were coded as clauses with adverbials and relative clauses that provide temporal‐locative‐causal information, meaningful conjunctions combining two or more clauses, if‐then statements, and reported speeches. We analysed the proportion of complex clauses to total clauses. The same calculation was used for both story and cartoon tasks. Two researchers coded 20% of the data for the reliability of the coding for each task, and each coder discussed where they were different. The remaining data was coded accordingly. The interrater reliability was assessed using intraclass correlation analyses (ICC) with absolute agreement. The results showed good reliability for both picture‐story (ICC = .82, *p* < .001) and animated video‐story (ICC = .82, *p* = .010) (Koo & Li, 2016).

##### Narrative complexity

For each child, a criterion‐referenced scoring method from the Test of Narrative Language—Second Edition (TNL – 2, Gillam & Pearson, 2017) was used for each story in storytelling. The same scoring method was used for each child's narrative complexity regarding the cartoon task. Children should have produced coherent narratives that included dialogues and temporal and causal relationships, using necessary linguistic elements without grammatical errors (see Appendix 1 for details). There were six categories: stating time relationship, causal relationship, using grammatically correct sentences, including dialogues, statements making sense, and the story being complete. Children could get 0 as minimum and 2 as maximum for each category. They would get a minimum of 0 and a maximum of 12 as a narrative complexity score. Another researcher coded 20% of the data for a reliability check for each task. The interrater reliability was assessed using intraclass correlation analyses (ICC) with absolute agreement. The results showed moderate reliability for picture‐story (ICC = .67, *p* = .007) and good reliability for the animated video‐story (ICC = .86, *p* < .001) (Koo & Li, 2016). Each coder discussed where they were different, and the remaining data was coded accordingly. We used the same coding scheme for both tasks since the narrative complexity scores represented general knowledge about stories; applying it to stories presented with different elicitation methods seems reasonable.

## RESULTS

We conducted an ANCOVA to examine whether PT children differed from FT children in their expressive vocabulary skills, controlling for age. Because we had two picture‐story tasks, we conducted Pearson correlation analyses to see whether we could average the scores from the two tasks. Pearson correlation analyses revealed that the total number of words, linguistic complexity, and narrative complexity scores in Picture Story 1 significantly correlated with those in Picture Story 2. Thus, we averaged these scores across Picture Story 1 and Picture Story 2.^2^ Then, we conducted six ANCOVA analyses to compare the total number of words, linguistic complexity, and narrative complexity for picture and animated video‐story tasks, controlling for age. We implemented Bonferroni corrections by classifying the analyses according to the number of comparisons conducted within each presentation mode. The *p*‐values for both picture and animated video narratives were calculated by dividing 0.05 by 3. Consequently, the adjusted significance level was established at 0.017. Since not every child completed all tasks, we handled the missing data by excluding cases pairwise.

First, we asked whether PT children differed in their general expressive language scores. Controlling for age, we found that PT children had lower expressive vocabulary scores than FT children: *F*(1, 64) = 4.70, *p* = .034, partial *η* ^2^ = .068 (see Table 1). Age was not a significant covariate, *F*(1, 64) = 1.92, *p* = .171, and there was no interaction between age and neonatal status, *F*(1, 63) = 0.125, *p* = .725.

Then, we asked whether PT and FT children differed in the total number of words and linguistic complexity as the microstructure dimension of the narratives and narrative complexity as the integration score of the linguistic and pragmatic aspects in the picture‐story. For the total number of words, we conducted a Quade Non‐Parametric ANCOVA since the normality assumption was not met.^3^ Controlling for age, results showed a main effect of neonatal status *F*(1, 62) = 4.79, *p* = .032. PT children narrated longer stories than their FT peers (see Table 1). However, this finding did not survive the Bonferroni correction. For linguistic complexity, again, we conducted a Quade Non‐Parametric ANCOVA and found no significant main effect of neonatal status *F*(1, 62) = 0.78, *p* = .382, controlling for age. For narrative complexity, we conducted ANCOVA and showed no significant main effect of neonatal status *F*(1, 62) = 3.41, *p* = .070, controlling for age. Age was not a significant covariate and nor was there any interaction with age *F*s < 0.77, *p*s > .05.

Then, we asked whether the animated video story would yield similar results. Unlike the picture‐based story, controlling for age, Quade Non‐Parametric ANCOVA^4^ showed no main effect of neonatal status on the total number of words, *F*(1, 45) = 1.76, *p* = .191. Similar to picture story, Quade Non‐Parametric ANCOVAs showed no main effect of neonatal status on either linguistic complexity or narrative complexity, *F*(1, 45) = 2.44, *p* = .125, *F*(1, 45) = 1.05, *p* = .298, respectively.

## DISCUSSION

We investigated the link between neonatal status and narrative performance in the microstructure dimension (i.e., the total number of words and linguistic complexity) as well as the integration of the microstructure and macrostructure dimensions (i.e., narrative complexity) in preschool‐aged children through two presentation modes. First, we asked whether PT and FT children differed in their general assessment of expressive vocabulary scores. Next, we asked whether there would be group differences between PT and FT children regarding the three dimensions of narratives (total number of words, linguistic complexity, narrative complexity). Lastly, we asked whether PT and FT children performed similarly across picture‐story and animated video‐story tasks with possible differences in their demands.

First, supporting earlier findings (Stipdonk et al., 2020), we showed that PT children had lower expressive vocabulary scores (i.e., the number of words they produce in daily life) than their FT peers. Although Luu et al. (2009) reported that the gap in receptive language skills (i.e., comprehension of spoken words) between PT and FT children decreased over time, PT children may still have lower expressive vocabulary skills. However, expressive vocabulary may not accurately reflect language skills in real‐life situations. One aspect of using language in natural contexts is narrative generation.

Regarding narrative generation, our findings supported the hypothesis that, although some aspects might have varied, PT children generated narratives that were comparable to those of their FT peers. Overall, both FT and PT children had difficulty generating narratives and received low scores (see Appendix 2 for examples). Nevertheless, our results showed comparable performance levels, particularly in linguistic complexity and narrative complexity, regardless of the presentation mode of the narratives. However, in the picture‐story mode, PT children tended to produce longer narratives. These results support earlier findings, indicating that PT children possess lower expressive vocabulary skills and may exhibit different performance patterns across the microstructure and macrostructure dimensions of the narratives. We also extend this conclusion to younger children, particularly 4‐year‐olds (Crosbie et al., 2010; Smith et al., 2014; Stipdonk et al., 2020). Nevertheless, our findings diverge from earlier research concerning the microstructural dimension. In contrast to their results, PT children produced sentences of equivalent complexity in our study.

PT children's lower expressive vocabulary scores but comparable narrative skills at 4 years of age might indicate the cascading effects of child‐ and environment‐related factors (Oakes, 2023; Oakes & Rakison, 2019), particularly parental multimodal language input (Göksun et al., 2025). For instance, due to their limited motor skills (Kobaş et al., 2023), PT infants might explore fewer objects, receive less language input from their parents, and know fewer words (M. Kobaş, E. Demir‐Lira, İ. Akman, & T. Göksun, unpublished data). Moreover, parents might adapt their input to the perceived language skills of their PT infants, providing less complex examples of language use (Fusaroli et al., 2019; Kızıldere et al., 2022). Supporting this argument, Özdemir et al. (2024) reported that PT children received fewer words in their parental input compared to their FT peers at 14, 20, and 26 months. Thus, PT children might not have the same opportunities to develop comparable vocabulary sizes with their FT counterparts. Furthermore, parents are not always accurate in their perception of their children's word knowledge and tend to overestimate their children's skills, which is associated with how they tailor their vocabulary but not the complexity of their language input (Ertaş et al., 2024). Thus, PT children might end up with lower expressive vocabulary knowledge due to the less optimal input they receive, but other aspects of language (e.g., linguistic complexity, narrative complexity) could remain comparable with FT children due to their ability to benefit more from stimulating environments (Demir‐Lira & Göksun, 2024).

Our results may suggest that PT children have caught up with their FT peers in narrative generation. However, would they perform similarly across picture‐story versus animated video‐story that may have different task demands? Because earlier studies observed performance differences across presentation modes in typically developing children (e.g., Diehm et al., 2020), our third question inquired whether neonatal status was associated with narrative performance similarly across the two narrative presentation modes (i.e., picture‐story vs. animated video‐story). Although we found similar performance trends across picture‐ and animated video stories, modest differences were observed between PT and FT children in their narrative length in the picture‐story. It has been suggested that PT children might be more prone to talk with repetitions or corrections to produce comparable narratives with their FT peers due to their limited expressive vocabulary (Crosbie et al., 2010). In line with the developmental cascades approach (Oakes, 2023; Oakes & Rakison, 2019), our findings suggest that PT children might use adaptive strategies to generate narratives depending on the presentation modes.

Despite both presentation modes being narrative generation tasks, they may or may not tap into different skills considering their structures and requirements. As was the case in earlier findings (e.g., Diehm et al., 2020; Richardson, 2024; Tibbets, 2022; Willis, 2022), the static (picture‐story) vs. dynamic (animated video) nature of our two stimuli would have differential effects on narrative generation. During the picture‐story task, children had the pictures in front of them while generating the stories, experiencing a cognitive load related to language production, whereas, during the animated video‐story task, they had to first watch a wordless cartoon and then generate a narrative, which may have increased their long‐term memory load. Consequently, the picture‐story may impose a greater demand on working memory, as it requires children to process images while simultaneously generating narratives. In contrast, the animated video‐story appears to engage more with long‐term memory, to which both FT and PT children in our sample appear to be equally affected. However, it should be noted that fewer children completed the animated video‐story compared to the picture‐story. Therefore, our sample size would lack the power to detect any differences. Additionally, the difference we found between PT and FT children in the picture‐story was modest and requires replication. All children in our sample may have struggled to generate narratives in the animated video format, indicated by lower scores in linguistic and narrative complexity, along with the number of children who did not complete the animated video‐story. Processing, recalling, and producing narratives featuring multiple protagonists and events may have led all children in our sample to talk longer only in the animated video‐story. Supporting this view, Luu et al. (2009) reported an increase in irrelevant talk to the story to maintain the flow of conversation while retrieving information. Moreover, memory‐intensive tasks (e.g., our animated video‐story) can result in simpler linguistic structures (Adams & Gathercole, 2000). Thus, we speculate that narrative generation in a task that included memory retrieval may have decreased both PT and FT children's focus on vocabulary richness and diversity.

Our study differed from the earlier work on narrative skills for its focus on narrative generation, as opposed to narrative retelling (e.g., Demir et al., 2015; Stipdonk et al., 2020). In contrast, we used two narrative generation tasks: picture‐story and animated video‐story. In narrative retelling, children are provided with a verbal narrative with its organized structure, and it relies on the extent to which children can use the linguistic input they receive, which can sometimes depend heavily on their memory capacity; in narrative generation, on the other hand, they need to mentally organize and connect the events with necessary linguistic elements. Thus, narrative generation might better reflect the ability to spontaneously organize narratives, which is a central aspect of narrative skills (e.g., Kunnari et al., 2016). Unfortunately, our stimuli were not matched in content, the number of episodes within the elicitation method, and their cognitive demands, which is a limitation of our study. Since our stimuli were not matched, we did not directly compare the two modes in terms of children's narrative scores. Rather, we focused on comparing children in each task and looked at whether the difference between children showed the same pattern across presentation modes. Thus, this limitation does not apply to the differences found between PT and FT children in our study. Future studies should create controlled stimuli to map individual differences and differential pathways related to both environmental and child‐related factors that may result in generating complex narratives despite lower linguistic skills in PT children. Using different stories may elicit performance differences in linguistic and narrative complexity. Therefore, careful matching of the story stimuli and their narrative complexity (e.g., sequences of events) would be advisable in future studies. Additionally, it may be worth assessing additional language and/or cognitive abilities, such as grammar or memory skills, outside of the narrative context to assess how these may interact with narrative generation.

In sum, we found that PT children had lower expressive vocabulary skills but were not different regarding linguistic or narrative complexity in either picture story or animated video story. However, in the picture story, PT children had a slight tendency to generate longer narratives than their peers. Our results may imply that children following different developmental trajectories resulting from PT birth might overcome the cascading negative effects of PT birth on narrative generation as early as 4 years of age.

## AUTHOR CONTRIBUTIONS

**İbrahim Akkan:** Conceptualization; methodology; formal analysis; writing – review and editing; writing – original draft; validation; visualization; data curation. **Şeref Can Esmer:** Conceptualization; writing – original draft; writing – review and editing; methodology. **Işıl Doğan:** Conceptualization; methodology; investigation; writing – original draft; writing – review and editing. **Aslı Aktan‐Erciyes:** Methodology; writing – review and editing; writing – original draft; supervision. **Ö. Ece Demir‐Lira:** Writing – original draft; writing – review and editing; supervision; methodology. **Tilbe Göksun:** Writing – review and editing; writing – original draft; project administration; resources; supervision; funding acquisition.

## CONFLICT OF INTEREST STATEMENT

We have no conflict of interest to disclosure.

## Data Availability

The data file and study analysis code will be available to share upon request from the corresponding author. This study was not preregistered.

## Footnotes

^1^
Maternal education was measured in terms of years, gestation week was collected in terms of weeks and birth weight was collected in terms of grams.

^2^
The correlations between two picture‐based stories for the total number of words, linguistic complexity, and narrative complexity were *r* (61) = .29, *r* (59) = .46, *r* (61) = .45, all *p*s < .05, respectively.

^3^
Shapiro–Wilk tests showed that in the picture‐story, the distributions of the total number of words and linguistic complexity departed significantly from normality (*W* = 0.95, *p* < .01, *W* = .94, *p* < .05, respectively).

^4^
Shapiro–Wilk tests showed that in the cartoon‐story, the distributions of all variables departed significantly from normality (*W* = 0.91, *p* < .01, *W* = .94, *p* < .05, *W* = .87, *p* < .05, respectively).

## APPENDIX 1 NARRATIVE COMPLEXITY CODING

**Table:**

Narrative complexity scoring			
	Score	Examples in Turkish	Examples in English
Temporal relationship			
No time relationship stated	0	—	—
Uses and or then	1	Çocuk geç kalıyor **sonra** elini yıkadı	The child is being late **then** he washed his hands
Uses one or more time adverbials	2	Önce, sonra, (verb) + dığında, (verb) + ınca	Before, after, when, as he was
Causal relationship			
No causal relationship	0	—	—
One causal relationship	1	Otobüsü yakalayama **+ dığı için** de öğretmeni ona kızmış	**Because** he missed the bus, his teacher was angry with him
Two or more causal relationships	2	Çünkü, (verb) + dığı için, böylece, bundan dolayı, sonuç olarak	Because, since, so, therefore, as a result
Grammatically correct sentences			
Three or more grammatical errors	0		
One or two grammatical errors	1	Önce çocuk kalkmış. Sonra okula **gitmesini** anlamış	First the boy woke up then he understood **his going** to the school
No grammatical errors	2	—	—
Indicates dialogue			
No dialogue	0		
Diologue for one character	1	Annesi “hı hı” demiş	His mother said “huh huh”
Two characters talk to each other	2	Öğretmeni “niye geç kaldın” demiş. Çocuk da demiş ki “otobüs dolmadı”	His teacher said “why are you late” and the boy said “the bus was not full”
Story makes sense			
Two or more sentences does not make sence or out of the sequence	0	Okula geç kaldığı için otobüs dolmamış	Because he was late to the school, the bus was not full
One statement does not make sense or out of the sequence	1		
All statements make sense	2		
Story is complete or complex			
Incomplete—missing a beginning, actions or an ending	0		
Complete—has a beginning, middle, endinfg	1		
Complex—goes beyond what is pictures	2		

*Note*: Bold words represent the examples and their English translations.

## APPENDIX 2 NARRATIVE EXAMPLES

**Table:**

An example of a child with a low narrative complexity score							
	Temporal relationship	Causal relationship	Grammar	Dialogue	Statement makes sense	Complete story	Total
	1	0	2	0	0	0	3
A boy was living with his mom			No grammar mistake				
Then the boy ran away from his mother	1						
Then							
Did something							
Did something							
Did something					−1		
What is he doing here?							
He fell, the game became water					−1		
The fishes have gone to their houses					−1		
Then							
His mother did a thing							
Aship way					−1		

An example of a child with a high narrative complexity score							
	Temporal relationship	Causal relationship	Grammar	Dialogue	Statement makes sense	Complete story	Total
	2	0	2	0	1	1	6
He is eating the food			No grammar mistake				
What are they doing?							
Uhm she is building a ship							
How is this possible?							
Letter					−1		
What happened							
She fell							
Yes what happened here?							
A ship							
Then it fell and it is broken	1						
Then what did they do?							
Then they ate							
After that they rebuilt the ship	1						
